# The first pelvic examination: A rite of passage for the women. A qualitative study about French women

**DOI:** 10.1080/13814788.2020.1760243

**Published:** 2020-05-13

**Authors:** Elodie Million, Amandine Yvon, Agnès Oude-Engberink, Pierre Mares, Philippe Serayet, Sylvain Pavageau, Bernard Clary, Béatrice Lognos

**Affiliations:** a Department of Family Medicine, University of Montpellier, Montpellier, France;; b Department of EA 4556 Epsylon laboratory, University of Montpellier, Montpellier, France;; c Department of CEPS Plateform, University of Montpellier, Montpellier, France;; d Maison de Santé Pluriprofessionnelle Avicenne, Cabestany, France;; e Department of Gynecology and Obstetrics, Hospital of Nîmes, University of Montpellier, France

**Keywords:** Pelvic examination, woman, prevention, qualitative research, patient-centred care

## Abstract

**Background:** French general practitioners (GP) and gynaecologists can make use of recommendations when performing a patient’s first pelvic examination. The indications and techniques for this examination are clear. The relational aspects and experience of the patients have been dealt with little.

**Objectives:** To analyse and understand the experience of French women during their first pelvic examination to propose practice recommendations based on their experiences.

**Methods:** Qualitative semi-structured interviews was conducted with 13 French women aged 18–30 years recruited from the surgery of a general practitioner using the snowball method. The data were analysed using an inductive method.

**Results:** The first pelvic examination was considered an indispensable rite of passage into adulthood and the life of a woman. They wanted a preparation for a consultation devoted to the first pelvic examination, with a time that is adapted to each woman. A patient-centred practitioner was more important than the pelvic examination itself.

**Conclusion:** Women requested for a general practitioner or a gynaecologist with a deeper understanding of a woman’s experience to perform their first pelvic examination. We propose practical recommendations: the following 3 phases for the consultation: before the pelvic examination where the women and the practitioners may get to know one another; during the examination, which would involve the technical aspects and the associated procedures; and after the examination, where the patients and the practitioners review the experience and discuss prevention.

KEY MESSAGES

- Practitioners must know that women do not consider their first pelvic examination as another medical examination.
- This intimate examination is considered an important rite of passage to adulthood and life as a woman.
- The experience of their first pelvic examination will impact their subsequent gynaecological follow-up.

##

KEY MESSAGES

- Practitioners must know that women do not consider their first pelvic examination as another medical examination.
- This intimate examination is considered an important rite of passage to adulthood and life as a woman.
- The experience of their first pelvic examination will impact their subsequent gynaecological follow-up.

## Introduction

A pelvic examination is performed in most gynaecological consultations. It is more intrusive than other medical examinations. In France, the first pelvic examination is often performed before the woman is of the eligible age to undergo the first Pap smear. For women, it can be a source of worry because it requires them to show a very intimate part of their bodies [1].

Practitioners should seek clear and practical recommendations on performing the first pelvic examination to make it as comfortable as possible and to decrease unpleasantness for a woman. The first pelvic examination technically should not be different from subsequent examinations. However, the first pelvic examination requires a specific communication approach with an appropriate explanation time. These conditions would make it possible to optimise the experience of this examination and therefore allow for a better experience in subsequent gynaecological examinations.

In France, the French National College of Gynaecologists-Obstetricians (Collège National des Gynécologues-Obstétriciens Français [CNGOF]) has proposed providing support material for performing a pelvic examination [2], but this tool does not deal with the relational aspect or gaining the patient’s trust. These technical recommendations are insufficient to optimise the experience of the first pelvic examination.

The international literature contains practical recommendations for healthcare professionals conducting a first-time pelvic examination on patients. These recommendations are found primarily in North American journals [3–10] and are mostly based on practitioners’ experience.

A few international studies conducted from 2003 to 2011, which were essentially quantitative in nature, aimed to analyse the experiences of women during their first pelvic examination [11–16]. These studies confirmed the importance of the relationship between a woman and a practitioner during the first pelvic examination, and the importance of the information received.

The first pelvic examination has been associated with a negative experience with anxiety in 50%–60% of women and pain in 30%–40% of women [13]. Women, especially young women, reported their fear of being naked, as well as their fear of the speculum [15].

Studies also highlighted factors associated with discomfort during a pelvic examination and a negative experience [11, 14]. These factors included poor contact with the practitioner, no permission requested by the physician to carry out the examination on the patient, a male practitioner, a youthful patient, the patient could not interrupt the examination, and a difficult life history. Moreover, unpleasant experience of the first examination was associated with a significant impact on subsequent sexual experiences of the patients [13].

The experience of the first pelvic examination could be decisive for women’s follow up. Moreover, practical recommendations cannot address the technical aspects and cannot be proposed only based on the experience of the practitioners.

The lack of French studies, compared to the more abundant international literature on the first pelvic examination, remains a concern. A study of the experience of French women regarding their first pelvic examination could enrich international data on the subject.

This study aimed to analyse and understand the experience of women during their first pelvic examination. This global approach [17–19] could allow us to propose practice recommendations for the first pelvic examination, based on women’s experiences. These recommendations should be proposed for international healthcare professionals.

## Methods

### Study design

This qualitative study used aspects of the Grounded Theory method. Two researchers performed this qualitative study within a global approach to the women’s lived experience. Semi-structured interviews were conducted using an interview guide focussing on lived experiences. The researchers adopted a phenomenological attitude and questioning (e.g. ‘what can you tell me about…?’, ‘What about the practitioner?’).

An inductive content analysis was conducted to allow the conceptualising categories to emerge. This analysis, based on Grounded Theory research, used the process of constant comparison between the text and categories to increase the categories’ level. In this study, our goal was not to theorise but to explore and describe the lived experiences of women [17–19].

### Setting

The women in our study were initially recruited from the private surgeries of three general practitioners.

In France, if a woman needs a pelvic examination, she can choose between a general practitioner, a gynaecologist or a midwife. They work in a private surgery practice or in a medical centre, called ‘family planning’ (a French organisation with contraception and prevention goals for sexual health). Women are commonly offered a pelvic examination during a gynaecological visit. There is no separate area for undressing in each examination room; sometimes, just a screen is provided [1].

### Selection of the participants

#### Selection criteria

The participants were included if they were between 18 and 30 years old and had already experienced a pelvic examination.

#### Exclusion criteria

We decided to exclude women over the age of 30 because their memories could be vague, and women under the age of 18 years for ethical reasons.

#### Sampling

The women were approached during visits to a French general practitioner’s surgery. One general practitioner included women. She was working in three surgeries in two French areas (Languedoc-Roussillon and Aveyron).

We started with a purposive sample (i.e. a targeted sampling of women who were competent to answer the research questions). This purposive sample was completed by a snowball sampling of women designated by the first sample and who were known to be competent to answer the research questions [20]. Women recruited by the snowball method were contacted by telephone. The snowball method allowed us to complete the purposive sample, which was otherwise too homogenous.

The participants were selected to obtain variance in their characteristics and experiences of the first pelvic examination. All women who were approached agreed to undergo the interview. No payment or financial compensation was proposed.

### Interview procedure

The interviews were carried out between June and December 2014. The interviews lasted between 18 and 32 min, with an average of 27 min. The interviews took place in an area chosen by the women, generally their homes, or the surgery of their general practitioner. At the beginning of the session, each participant completed a questionnaire that asked about age, socioeconomic group, marital status, number of children, and home address. The investigator (YA) was a general practitioner. She maintained a position of openness and empathy to facilitate discussion about these intimate matters [3,18].

The semi-structured interviews were based on an interview guide (Table 1) comprising open, as well as probing questions. This guide needed to be both adaptable and flexible [17,21]. The research team devised the guide based on data from the literature. It was modified and enhanced after the first two test interviews. These two interviews were shorter than the preceding interviews but were analysed and maintained because of significant data. Data collection and analysis were managed simultaneously, as in any qualitative research. This analysis allowed the researcher to adapt her discourse and reword it to complete the analysis of a previous interview.

**Table 1.** Interview guide.

I have introduced myself. Could you now tell me about yourself?
*Probing questions: Who are you? What about your job? What about your family?*
To continue, could you tell me about your medical consultations?
*Probing questions: How does it occur?*
More specifically, what can you tell me about your gynaecological consultations?
*Probing questions: where was or is it managed? What about the practitioner(s)?*
Can you tell me about your first pelvic examination?
*Probing questions: when was it? What about the practitioner? How have you been prepared for it ?*
What was your experience of this examination?
*Probing question: what kind of emotions did you experience?*
Before this first pelvic examination, did you have the opportunity to talk about it (with those close to you, doctors…)? Could you tell me about this discussion?
*Probing question: What about talking with a practitioner before the examination?*
Could you tell me how you imagined this examination would be?
*Probing question: what could you say to me about the difference between what you expected and what you lived?*
You have told me about your first pelvic examination, your experience and how you anticipated it. How has all influenced your consultations since then?
*Probing questions: what was the influence on your practitioner choice? On your next pelvic examination?*
Thank you for talking to me about your experience. To conclude, could you tell me, according to your history, how, in an ideal world, should this pelvic examination be presented and carried out?

The interviews were recorded on a voice recorder and transcribed word-for-word. The investigator offered to communicate final results of this study by mail allowing participants to comment on the results and thereby increasing the understanding of their experience. However, no participants asked for the results.

### Data analysis

No analysis software was used. The two investigators performed a triangulation of the analysis with inductive content analysis, using a process of constant comparison between the text and categories. This approach was justified by aiming to explore the personal experiences of the women, and to understand their representations [19] (Table 2).

**Table 2.** Analysis steps.

Accurate transcription of the recordings
Identifying the most relevant elements of the pre-existing context
General intuitive reading, followed by targeted reading
Dividing text into meaning chunks and then into the first themes
Identifying all indexical, textual and contextual signifiers for a first categorisation
Categories increase in level of generalisation through constant comparison
Characterization of conceptualising categories
Arranging those categories according to their logical inter-relationships to construct the meaning of a phenomenon

**Table 3.** Characteristics of the participants.

	Age	Socio-professional group	Marital status	Number of children	Home environment at the time of the examination	Age of their first pelvic examination (years old)
E1	27	Intermediate profession^a^	Common-law couple	1	Semi-rural	17
E2	28	Intermediate profession^a^	Married	1	Urban	20
E3	26	Intellectual profession^b^	Common-law couple	1	Urban	16
E4	24	Intermediate profession^a^	Single	0	Urban	16
E5	25	Employee	Common-law couple	0	Urban	18
E6	28	Intermediate profession^a^	Common-law couple	0	Semi-rural	14
E7	20	Student	Single	0	Urban	18
E8	26	Intellectual profession^b^	Single	0	Semi-rural	16
E9	28	Intermediate profession^a^	Common-law couple	1	Urban	14
E10	23	Student	Married	0	Rural	17
E11	22	Employee	Single	0	Urban	18
E12	26	Intermediate profession^a^	Common-law couple	0	Urban	16
E13	26	Intermediate profession^a^	Common-law couple	0	Urban	16

a Intermediate profession: Teacher, Social worker, Foreman ….

b Intellectual profession: Lawyer, Doctor, Professor, Engineer….

### Analysis example in the supplement

Interviews were performed until data saturation, which was achieved when a new interview did not produce new data to increase the level of categories. This effect occurred after 11 interviews. Two additional interviews were conducted to confirm data saturation.

The qualitative approach explored data ‘as it was’ expressed in the text without abusive interpretation to respect the trustworthiness of the data. Therefore, the interview guide was supported by a consensus between the researchers. The interviewer was trained in the phenomenological-inspired interview to obtain the lived experience. Comparing results to the literature allowed us to confirm the transferability (i.e. external validity) of the results in other contexts.

### Ethics

The purpose of the study, course of the interviews, registration, rules of anonymisation and confidentiality were recapped to the women before they began an interview. We recorded their responses while respecting the women’s’ anonymity and the possibility of talking about intimate matters. The protocol was approved by the research board of DUMG-Montpellier University (Montpellier, France). The French Ethical Research Committee deemed approval unnecessary because of the nonpharmaceutical biomedical research nature of this work (article L. 1123-7 du code de la santé publique).

Participants were informed that they could interrupt the interview and withdraw from the study at any time. Each woman confirmed by oral agreement to participate. A written document was then signed by the woman and the researcher.

## Results

### Characteristics of the participants (Table 3)

The 13 interviewed women were 20–28 years old. Two were married, seven were common-law couples, and four were single. Four women had one child and nine women had none.

### Analysis

Three conceptualising categories emerged from the analysis. We propose a synthesis of these three categories and their subcategories (Table 4).

**Table 4.** Categories and sub-categories.

Conceptualising category	Subcategory
The first pelvic examination is an indispensable rite of passage into adulthood and the life of a woman	Young women experience the pelvic examination as a transition into a new stage: from adolescence to a woman’s life.
	The first pelvic examination allows women to leave childhood behind and become adults
	The relationship between the woman and her mother (experience sharing, conflicts) can have an impact on their experience of their first pelvic examination: a good mother-daughter relationship improves this experience.
Preparation is necessary for a consultation devoted to the first pelvic examination, with a period of time that is adapted to the woman	The consultation was divided by the women into 3 distinct phases
	1st: The meeting phase between the woman and the practitioner
	2nd: The examination phase should be as quick as possible, but also gentle and painless.
	3rd: The conclusion phase of the consultation was necessary for reassuring the young women
	The discussions before and during the first pelvic examination were very important
	The women’s biographical aspects at the time of their first pelvic examination made each consultation unique
A patient-centred practitioner more important than the pelvic examination itself	Women wanted their choices to be respected
	The practitioner must take into account the woman's vulnerability during her first pelvic examination
	The quality of the relationship between the woman and the practitioner was even more important

### Category 1: The first pelvic examination, seen as an indispensable rite of passage into adulthood and the life of a woman

Following their first pelvic examination, the women felt that they had left their childhood behind and that they had had ‘an initiatic rite’. Some of the women interviewed had had a traumatic experience during their first pelvic examination, but they retained their perception of attaining an important life stage that allowed them to reach adulthood and a woman’s life. This first gynaecological examination was a necessary passage, an initiation rite, for the women interviewed, whether the examination experience had been good or bad. However, a negative experience of this examination could complicate subsequent gynaecological follow up.

Finally, … I felt like a woman. That’s it, I am big enough for someone unknown to take care of me at that [intimate] level. … I was a little proud to have done that. I thought: now I am no longer a child. (E1)

Women wanted the practitioner to consider them as adults, especially when they were very young, at their first gynaecological examination or when their mother accompanied them. Thus, young women would have experienced the presence of the mother as being harmful to the quality of the relationship with the practitioner, especially if the mother-daughter relationship was complicated. In these situations, the practitioner sometimes talked to the mothers who took the lead in the discussions. Young women expressed that being in the position of a child was the opposite of what they wanted for their first pelvic examination. However, some women reporting good mother-daughter relationships considered the presence of the mother during the consultation as reassuring and positive.

I remember it was my mother who answered the questions, I did not talk so much … She was mad at me for a bit because she did not think I could have a sexual relationship at that age… She was angry at me so the discussion was difficult… (E6)

I went with my mom; In fact, the question did not arise, I told her to come and she came … I had nothing to hide from her. (E10)

### Category 2: Preparation necessary for a consultation devoted to the first pelvic examination, with a time that is adapted to the woman

In this category, five subcategories emerged: three distinct phases in the consultation, the discussions before and during the first pelvic examination, and the women’s biographical aspects at the time of their first pelvic examination.

- The meeting phase between the woman and the practitioner.
  Women feel the need to get to know the practitioner who will perform their first pelvic examination. Although the women expected the practitioner to ask questions, an in-depth medical interrogation would have been regarded as too inquisitive with the practitioner not personalising the consultation or seeking to get to know the woman behind the patient. On the contrary, the absence of exchange before carrying out the examination increased the fear it provoked.
- *The discussion is critical to make an examination.*
  It is necessary to prepare [the woman] to what will happen. I did not get any accompaniment, … I was dragged by the scruff of my neck and ‘bim’! Upon the table. (E9)
- The examination phase should be as quick as possible, but also gentle and painless.
  The practitioners should be reassuring without trying to modify their discourse, which should remain medical, to guide the woman through the examination.
  It’s someone who is rather sweet … I think I could have experienced a very bad examination if the person I had in front had been abrupt in his actions … (E4)
- *The concluding phase of the consultation was necessary for reassuring the young women*.
  This phase was the time for an effective discourse on prevention (contraception, sexually transmitted diseases, etc.), but was only very rarely present in the consultations of the women interviewed.
- Discussions before and during the first pelvic examination were very important.
  The women requested preparation before the examination with their mothers and professionals to improve their experience. The women had often conversed with their mothers before their first pelvic examination. Thus, they were aware of the importance of gynaecological follow-up.
  *I knew my mother did not like it! So, it was stressing me because she told me it was unpleasant. (E3)*
  I knew that if there was a problem, I could talk to my mother, that there was no problem. We’ve always talked a lot about all that … (E8)
  The feeling of ‘enough time’ taken by the practitioner varied considerably from one woman to another and impacted the experience of the first pelvic examination. The women needed to feel support during their first pelvic examination. They needed to have had enough time to feel like an individual to the practitioner and not just another gynaecological visit.
  So … she spent time, yes, but not necessarily for me … I did not find that she took care of myself … It was fast what … I was just another smear. (E5)
- *The women’s biographical aspects at the time of their first pelvic examination made each consultation unique.* The women needed the practitioners to personalise their care, taking their individual stories into account: age, relationship with their mother, sexual experiences, reason for the consultation, interactions before the examination, and the prejudices of the women regarding the examination, which was necessarily painful and traumatising, or prejudices about the gender of the practitioner.
  *That feeling of having unveiled all your life in the space of half an hour. (E12)*
  It was after my first sexual intercourse, I was 14 years old and I thought I was pregnant … So, I was very embarrassed, I was not in my place … And then, I was not ready for all that. (E6)

### Category 3: A patient-centred practitioner more important than the pelvic examination itself

The women agreed about preferring certain material considerations: a separate room for undressing, the possibility for partial nudity, a plastic speculum [the stainless-steel speculum had a very negative image for the women]:

it’s a bit old-fashioned, you know! (E5)

Women’s practical recommendations were important, but the quality of the relationship between the woman and the practitioner was even more so. Non-judgmental patient-centred practitioners reassured the women.

An examination that did not go well from a technical point of view was still considered positive if the quality of the patient-physician relationship was good.

The women felt vulnerable when recalling their first pelvic examination. They were afraid of the nudity, the pain, and the judgement.

Women wanted their choices to be respected: choice regarding the time of the examination, the degree of nudity, and whether or not to perform the examination.

I think everything is played before, because after, if she [the woman] is relaxed, the doctor can discuss … Yes, for me, everything is played before the doctor has touched the person … (E5)

## Discussion

### Main findings

This study aimed to understand how women experienced their first pelvic examination. The women assessed in our study (1) considered the first pelvic examination an indispensable rite of passage into adulthood and life of a woman; (2) considered that preparation was necessary for a consultation about the first pelvic examination. Women divided the consultation into three distinct phases: (a) the meeting phase between the woman and the practitioner; (b) the examination phase (‘quickly, gentle, painless’); and (c) the conclusion phase (reassuring the young women). Also, (d) the conversations before and during the first pelvic examination were very important. Moreover, (e) the women’s biographical aspects at the time of their first pelvic examination made each consultation unique. And (3) they also considered a patient-centred practitioner is more important than the pelvic examination itself.

### The first pelvic examination as a statutory transition: A possible theory

The purpose of our work was not to promote a theory, but our results are in line with the conclusions of Glaser and Strauss regarding statutory transitions. For these authors, who are the founders of the Grounded Theory, death, work, and marriage are statutory transitions that are accompanied by initiation rites [22]. In our study, women experience this pelvic examination as a ‘statutory transition’. While this finding may be linked to the young age of the women interviewed—a change in status from childhood to adulthood—it is not the only explanation. The statutory transition is also consistent with the intimate nature of this examination: allowing entry to a woman’s world, whatever the age of this first examination.

### Comparison with the literature

The international recommendations regarding the first pelvic examination are based on the experiences of practitioners [6]. Most recommendations are consistent with the expectations of the women interviewed in our study [4–10,22]. However, we would like to propose the addition of recommendations, based on women’s experiences.

A few international studies have analysed the experiences of women during their (first) pelvic examination. The conclusions of these studies produced concepts similar to those identified by the women in our study. French women had identical experiences to women elsewhere in the world, like Denmark, Turkey, Germany, Great Britain, and Sweden [11–16]. Previous studies and our study analysed women’s experience of their first gynaecological examination and found the following important concepts: the major status of the patient-centred practitioner, whom the woman has chosen, and the importance of individualised preparation and information during the examination. We confirm that our results can be extrapolated to other countries We also propose recommendations to countries that have a similar socioeconomic framework as that of France [4–16].

In the international literature, two aspects are described, which we did not find in our study: the gender of the practitioner and the sexual aspect of the pelvic examination. The women in the study by Gupta reported they preferred a female practitioner for a better experience of their first pelvic examination [14]. The women in our study accepted a male practitioner, female practitioner, or had no preference. This finding confirmed the maximum variation of experience of our sample. One of our conceptualising categories showed the importance of respecting the patient’s choice regarding the gender of the practitioner, but there was no clear link between the doctor’s gender and the patient’s experience.

In Grundström’s qualitative study [16] using phenomenological analysis, the sexual aspect of the examination was mentioned by the women: without receiving information during the pelvic examination, they could not be certain of the examiner’s intentions, especially if the examiner was male. The women in our study did not mention this sexual aspect. They confirmed that the pelvic examination should be as technical as possible with information given regarding what the practitioner is doing to limit the embarrassment that this intimate examination can generate. In Grundstörm’s study, the possible sexual connotation of the pelvic examination seemed to be related to male practitioners, whereas in our study women expected the same safeguards from practitioners during a pelvic examination whether they were male or female.

### Strengths and limitations

In our study, credibility was respected by the methodological choices. The inductive content analysis with constant comparison between the text and the categories was consistent with the objective of our study because it was relevant for assessing the experience of the women. For data significance, a triangulation of researchers was implemented, allowing for agreement on the interpretative inferences (thematization and categorisation). We can also confirm the credibility of our study based on the international criteria proposed by Guba and Lincoln [3], namely, the ‘prolonged engagement’ and ‘persistent observation’ was respected.

Qualitative research has long been adapted to exploring the experiences of women and their representations. Quantitative studies have already been conducted on this subject, but they do not allow such comprehensive analysis [11–14]. In our study, the qualitative method and especially aspects of the Grounded Theory allowed a deep understanding of the experience of women regarding their first pelvic examination. During the interviews, the researcher focussed on rewording to clarify an idea without guiding the woman’s responses. Each interview was analysed immediately to respect the qualitative method, and the researchers reached an agreement for analysis and interpretation. The triangulation of the analysis by two researchers, and the methodological coherence between the collection method and the aspects of Grounded Theory analysis, increased the solidity and credibility of our results. These findings may be transferable to similar settings, especially in countries with the same social or cultural structures, as confirmed in the international literature.

One of the investigators personally knew some of the participants and interviewed those women. To limit the bias associated with this, she exactly followed the same data collection method for all interviews. The analysis was done gradually and in the same time by both researchers. Investigators gave individual attention, with careful supervision, to the women interviewed when they knew them personally. The triangulation of the analysis by the two researchers ensured that the discourse of these women was not modified by the knowledge of the interviewer. On the contrary, in this case, the women interviewed talked more easily about this intimate examination and gave particularly rich interviews.

The choice of the age inclusion criterion (18–30 years) can be justified by the need for the participant to be able to remember the first pelvic examination. Older women would have been able to provide other insights and increase the variety in our sample. We would nevertheless have been faced with a risk of loss of information because of recall bias.

### Practical implications and future perspectives

In France, no recommendations or publications are available to raise the awareness of practitioners about the importance of this ‘initiatic rite’ and to suggest ways of making it the first pelvic examination more comfortable for the patient. A good experience is particularly important for the first examination, although the same precautions need to be taken for all pelvic examinations. To optimise the women’s experience of this examination, we envisage creating French recommendations similar to those used in other countries [3–10].

Our work adds new scientific knowledge. Each practitioner performing a woman’s first gynaecological examination should consider the following: women experience their first pelvic examination as an indispensable rite of passage into adulthood and the life of a woman. This information confirms the importance of this examination to the woman and it could impact their subsequent gynaecological follow-up. Through our work, practitioners should be able to rely on practical recommendations relating to the first gynaecological examination, based on women’s experiences. We can propose certain guidelines for practitioners performing a patient’s first pelvic examination, based on the women’s experiences of our study (Table 5).

**Table 5.** Proposed guidelines for practitioners performing a woman’s first pelvic examination based on women’s perceptions and expectations.

A patient-centred practitioner
Be empathetic and a good listener Never be, or seem to be, judgmental of either moral or physical aspects Prepare the examination and take however much time is necessary for the patient (several consultations if needed) Have the patient systematically allow the presence of any third party
Limit the embarrassment associated with modesty and the examination itself
Examine the woman in a room that is separate from the office Provide a separate area for undressing (separate room or behind a screen) Propose partial nudity (undressing in two stages, examination gown) Personalise the examination and how it is prepared with each patient Ask for the patient’s permission before examining her and accept her possible refusal Give women the opportunity to interrupt an examination in progress
Respect the three stages of the consultation
*Before the pelvic examination: time for getting to know one another and preparing for the examination* Show an interest in the patient (what she is, what she knows, what she fears and what she wants) Do not do a complete interrogation (too intrusive) Prepare the woman for the examination by explaining what will happen and what the purpose is
*During the examination: the technical aspect and accompaniment* Accompany each stage: warn the patient about what sensations she should expect before doing each act, and always explain why each act is necessary Perform the examination quickly, but gently and without causing pain Use a disposable plastic speculum and explain its role to the patient before the examination Reassure the patient whenever possible that the examination is normal Maintain a technical, medical discourse, without trying to create a diversion
*After the examination: the conclusion and time to talk about prevention* Always take the time to conclude the consultation, ensuring that the patient has fully understood and is on board with everything Talk about prevention only at this time for better efficacy

## Conclusion

Women have requested that they be better prepared for their first pelvic examination, which represents a major initiation rite for them, but is also a source of worry. The patient-centred approach is essential for ensuring that this examination goes well. Practitioners could be helped by the guidelines proposed in our study. These recommendations could be assessed in other qualitative and quantitative research projects. The aim would be to achieve acceptance of these recommendations by healthcare professionals and to facilitate their implementation.

## Data Availability

All the interviews are available at the General Practice Department of Montpellier-Nîmes, France, from Dr Elodie Million.
